# Evaluation of a New Spike (S)-Protein-Based Commercial Immunoassay for the Detection of Anti-SARS-CoV-2 IgG

**DOI:** 10.3390/microorganisms9040733

**Published:** 2021-03-31

**Authors:** Kirsten Alexandra Eberhardt, Felix Dewald, Eva Heger, Lutz Gieselmann, Kanika Vanshylla, Maike Wirtz, Franziska Kleipass, Wibke Johannis, Philipp Schommers, Henning Gruell, Karl August Brensing, Roman-Ulrich Müller, Max Augustin, Clara Lehmann, Manuel Koch, Florian Klein, Veronica Di Cristanziano

**Affiliations:** 1 Department of Tropical Medicine, Bernhard Nocht Institute for Tropical Medicine & I. Department of Medicine, University Medical Center Hamburg-Eppendorf, 20251 Hamburg, Germany; k.eberhardt@bnitm.de; 2 Institute for Transfusion Medicine, University Medical Center Hamburg-Eppendorf, 20251 Hamburg, Germany; 3 Institute of Virology, Faculty of Medicine and University Hospital Cologne, University of Cologne, 50935 Cologne, Germany; felix.dewald@uk-koeln.de (F.D.); eva.heger@uk-koeln.de (E.H.); lutz.gieselmann@uk-koeln.de (L.G.); kanika.vanshylla@uk-koeln.de (K.V.); maike.wirtz@uk-koeln.de (M.W.); franziska.kleipass@hhu.de (F.K.); philipp.schommers@uk-koeln.de (P.S.); henning.gruell@uk-koeln.de (H.G.); florian.klein@uk-koeln.de (F.K.); 4 Center for Molecular Medicine Cologne (CMMC), University of Cologne, 50931 Cologne, Germany; roman-ulrich.mueller@uk-koeln.de (R.-U.M.); clara.lehmann@uk-koeln.de (C.L.); manuel.koch@uni-koeln.de (M.K.); 5 Institute for Clinical Chemistry, Faculty of Medicine and University Hospital Cologne, University of Cologne, 50937 Cologne, Germany; wibke.johannis@uk-koeln.de; 6 Department I of Internal Medicine, Faculty of Medicine and University Hospital Cologne, University of Cologne, 50937 Cologne, Germany; max.augustin@uk-koeln.de; 7 German Center for Infection Research (DZIF), Partner Site Bonn-Cologne, 50931 Cologne, Germany; 8 Nierenzentrum Bonn, Godesberger Allee 26, 53175 Bonn, Germany; brensing.bonn@t-online.de; 9 Department II of Internal Medicine, Faculty of Medicine and University Hospital Cologne, University of Cologne, 50937 Cologne, Germany; 10 Cologne Excellence Cluster on Cellular Stress Responses in Aging-Associated Diseases, CECAD, University of Cologne, 50931 Cologne, Germany; 11 Institute for Experimental Dentistry and Oral Musculoskeletal Biology, and Center for Biochemistry, Faculty of Medicine and University Hospital Cologne, University of Cologne, 50931 Cologne, Germany

**Keywords:** pandemic, humoral response, neutralization, titer, protection, non-responder, coronavirus, antibody, COVID-19, sensitivity

## Abstract

**Background**: The investigation of the antibody response to SARS-CoV-2 represents a key aspect in facing the COVID-19 pandemic. In the present study, we compared the new Immundiagnostik IDK® anti-SARS-CoV-2 S1 IgG assay with four widely-used commercial serological assays for the detection of antibodies targeting S (spike) and NC (nucleocapsid) proteins. **Methods:** Serum samples were taken from an unbiased group of convalescent patients and from a negative control group. Sample were simultaneously analyzed by the new Immundiagnostik IDK^®^ anti-SARS-CoV-2 S1 IgG assay, by the DiaSorin LIAISON^®^ SARS-CoV-2 S1/S2 IgG assay, and by the Euroimmun anti-SARS-CoV-2 S1 IgG ELISA. Antibodies binding NC were detected by the Abbott SARS-CoV-2 IgG assay and by the pan-immunoglobulin immunoassay Roche Elecsys^®^ anti-SARS-CoV-2. Moreover, we investigated samples of a group of COVID-19 convalescent subjects that were primarily tested S1 IgG non-reactive. Samples were also tested by live virus and pseudovirus neutralization tests. **Results:** Overall, the IDK® anti-SARS-CoV-2 S1 IgG assay showed the highest sensitivity among the evaluated spike (S) protein-based assays. Additionally, the Immundiagnostik assay correlated well with serum-neutralizing activity. **Conclusions:** The novel IDK^®^ anti-SARS-CoV-2 S1 IgG assay showed high sensitivity and specificity, representing a valid option for use in the routine diagnostic.

## 1. Introduction

The investigation of the humoral response to SARS-CoV-2 represents a key aspect in facing the COVID-19 pandemic. Although neutralizing antibodies are considered to have an important protective role, the association between seropositivity and immunity, as well as the duration of protective humoral response are main questions of current research [1,2,3,4]. The U.S. Food and Drug Administration (FDA) declared a neutralizing titer ≥ 1:160 as sufficient for donation of convalescent plasma. However, the definition of an antibody titer conferring protection is still missing [5].

Virus neutralization (VN) assays, based on live virus or pseudovirus, are considered the gold standard to conclude on the presence and quantity of specific neutralizing antibodies. However, they are time intensive and require biosafety level 2 or 3 facilities [6]. Most routine diagnostic facilities instead make use of commercial serological immunoassays and high-throughput automated platforms [7]. Various methods for antibody detection are available, including chemiluminescence immunoassays (CLIA), chemiluminescent microparticle immunoassays (CMIA), electrochemiluminescence immunoassays (ECLIA), and enzyme-linked immunosorbent assays (ELISA). They can be divided into assays recognizing specific anti-SARS-CoV-2 antibodies against different antigens, including the spike (S) protein or components thereof (e.g., S1/S2 domains or the receptor-binding domain [RBD]) and the nucleocapsid (NC) protein [8]. Neutralizing antibodies mainly target the RBD domain located in the S1 domain of the spike protein [6,9]. Hence, S-protein-based assays might be considered more suitable as a surrogate for protection [10].

The number of studies providing data about the performance of various serological assays has massively increased. So far, the Roche Elecsys^®^, a pan-IG assay for the detection of anti-NC antibodies, was reported to be one of the most sensitive SARS-CoV-2 antibody detection assays [11]. However, its results correlated less with neutralizing titers than those of assays detecting anti-S antibodies [10].

Continuous evaluation of commercial assays targeting the S-protein is relevant for many reasons. The intensity of the antibody response can largely vary in asymptomatic and mild COVID-19 cases, and a relevant proportion of such patients apparently does not mount humoral response to SARS-CoV-2 that is detected by established commercial assays [12,13,14]. In addition, there are contradictory observations on the persistence of specific antibody levels over time in these groups [15,16,17,18,19]. In the case of anti-NC antibodies, more pronounced differences in sensitivity over time were already reported for some serological assays [10]. For this reason, it is of outstanding importance to investigate if non-detection is equal to absence of antibodies or just a result of less sensitive laboratory assessment methods. As a result, seroprevalence may be underestimated [20,21] and individuals carrying a SARS-CoV-2 B cell response are less likely to be detected [22,23]. So far, despite apparently significant rates of low- or non-responders determined by serological assays, re-infections with SARS-CoV-2 are still reported to be a rare event [24]. Based on these considerations, assay sensitivity could represent a decisive aspect for understanding the mechanisms underlying protective humoral responses. Furthermore, highly sensitive S-based protein assays could be relevant for defining time intervals for vaccine boosters, as well as for long term antibody response studies upon vaccination. Finally, the possibility to combine S- and NC-based proteins assays with similar high sensitivity and specificity will be increasingly required to answer the important question of SARS-CoV-2 infection despite active immunization.

Here, we evaluated the sensitivity and specificity of a novel S-protein-based commercial assay: IDK^®^ anti-SARS-CoV-2 S1 IgG by Immundiagnostik. Analyzed samples were obtained from a cohort of convalescent individuals, most of which had recovered from mild COVID-19 and were recruited at a German University Hospital after April 2020 [16]. We also investigated individuals with molecularly confirmed prior SARS-CoV-2 infection by real-time PCR but undetectable serum IgG antibodies as tested with a commercial S1 immunoassay. Additionally, the performance of the new assay by Immundiagnostik was compared to four other IgG qualitative immunoassays, including Roche Elecsys^®^ (anti-NC pan-Ig), Abbott (anti-NC IgG), Euroimmun anti-SARS-CoV-2 S1 IgG ELISA, and DiaSorin LIAISON^®^ (anti-S1/S2 IgG) (Table 1). Finally, the correlation between antibody levels and serum-neutralizing activity was investigated by live virus and pseudovirus neutralization assays.

## 2. Materials and Methods

### 2.1. Ethical Consideration

All subjects gave their informed consent for inclusion before they participated in the study. The study was conducted in accordance with the Declaration of Helsinki, and samples were collected and analyzed under protocols approved by the Institutional Review Board of the University of Cologne, Germany (16-054 and 20-1187).

### 2.2. Study Population

Serum samples were obtained from individuals with molecularly-confirmed prior SARS-CoV-2 infection recruited at a German University Hospital between April and September 2020. Most patients had recovered from mild COVID-19 and had not required hospitalization [16]. Some of the serological (excluding the Immundiagnostik test) and neutralization data used for comparison in this study have been partially presented previously in Vanshylla et al. [16]. In the current study, we investigated two subgroups of this cohort: group A included all convalescent patients with previous SARS-CoV-2 infection who attended the clinic between August and September 2020, regardless of their IgG ratio values. Group B included a group of individuals who attended the clinic between April and July 2020 and were tested IgG negative or borderline in our routine screening using the Euroimmun S1 IgG ELISA despite prior SARS-CoV-2 infection. Additionally, we included a group C composed of samples collected in early 2020 from individuals without any suspicion for SARS-CoV-2 infection, as well as serum samples obtained in 2019 prior to the SARS-CoV-2 pandemic as negative controls.

### 2.3. Establishment of A New SARS-CoV-2 Serological Test

**Figure 1:**
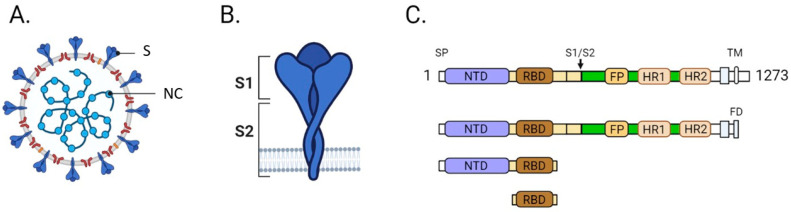
(**A**) Schematic drawing of the SARS-CoV-2 virus. Spike (S) and nucleocapsid (NC) proteins are highlighted. (**B**) Schematic drawing of the trimeric S-protein. (**C**) The domain structure of the S-protein is shown, and the three regions that were tested are depicted. All three versions contain the receptor binding domain (RBD). SP, signal peptide; NTD, N-terminal domain; S1, spike protein subunit 1; S2, spike protein subunit 2; FP, fusion peptide; HR1, heptad repeat 1 domain; HR2, heptad repeat 2 domain; TM, transmembrane domain; FD, foldon motif.

The novel assay IDK^®^ anti-SARS-CoV-2 S1 IgG and IgM was developed through a collaboration between Immundiagnostik and the University Hospital Cologne (M.K.). Serological tests mainly focus on the S or NC proteins from the SARS-CoV-2 virus (Figure 1A,B). As the S-protein might be the most critical protein for viral infection of target cells and expressed on the surface of the virion, different regions of the S-protein have been compared and evaluated for use in an ELISA kit. Three different regions, the S-protein ectodomain, the RBD, and a truncated S1 domain, have been recombinantly expressed in HEK293 cells and intensively tested (Figure 1C). The S1 truncated was chosen for further usage since it performed the best in different tests. According to Immundiagnostik, 7 out of 762 plasma samples, collected between 2017 and 2018, tested positive for the SARS-CoV-2 by ELISA. Furthermore, no cross reactivity to plasma probes for Adenovirus, Epstein-Barr Virus, Influenza A/B, HCoV-229E, HCoV-HKU1, HCoV-NL63, and HCoV-OC43 was detected. Additional information on the establishment of this assay are provided in the supplementary materials (Extended Material and Methods, Supplementary Materials Figure S1).

### 2.4. Serological Testing for the Detection of Anti-SARS-CoV-2 Antibodies

#### 2.4.1. Commercial Assays

Serum samples from groups A, B, and C were simultaneously analyzed by Immundiagnostik IDK^®^ anti-SARS-CoV-2 S1 IgG assay and four additional commercial assays for the detection of anti-SARS-CoV-2 antibodies (Supplementary Materials Table S1).

Anti-SARS-CoV-2 IgG targeting the S-protein were detected by the LIAISON^®^ SARS-CoV-2 S1/S2 IgG test (CLIA) on the LIAISON^®^ XL (DiaSorin, Vicenza, Italia), by the Euroimmun anti-SARS-CoV-2 IgG ELISA on the Euroimmun Analyzer I (Euroimmun Diagnostik, Lübeck, Germany), and by the IDK^®^ anti-SARS-CoV-2 IgG ELISA (Immundiagnostik AG, Bensheim, Germany) on the DYNEX DSX^®^ (Dynex Technologies, Chantilly, VA, USA). The two latter ELISA assays use the recombinant S1 antigen from of the spike protein (Figure 1).

NC protein-targeting antibodies were detected by the SARS-CoV-2 IgG assay provided by Abbott (CMIA) on the Alinity i (Abbott, Abbott Park, IL, United States) and by the pan-immunoglobulin immunoassay Elecsys^®^ Anti-SARS-CoV-2 (ECLIA) on the Cobas 8000 (Roche Diagnostics, Mannheim, Germany).

All assays were interpreted according to the manufacturers’ recommendations. IgG values by Immundiagnostik were capped at 3.8 OD at the upper end for analysis. In case of the assays by Euroimmun and DiaSorin, borderline results were counted as negative. However, we alternatively recalculated our findings counting borderline results as positive to evaluate if test performances would profoundly differ.

#### 2.4.2. Live Virus Assay to Determine SARS-CoV-2 Neutralizing Activity (LVN)

After inactivation by heating at 56 °C for 30 min, serum samples were diluted to 1:10 and 1:50 in DMEM (Dulbecco Dulbecco’s Modified Eagle’s Medium, Gibco, Dublin, Ireland) and mixed with 100 TCID50 (50% tissue culture infectious dose) of live virus (isolated from naso- and/or oropharyngeal swabs at the University Hospital Cologne using VeroE6 cells for infection and harvesting virus supernatants [16] to a volume of 100 µL). The virus-serum mixture was incubated for one hour at 37 °C. Afterwards, 50 μL of Vero E6 cell suspension (250,000 cells/mL) were added to each sample dilution. Cells were incubated at 37 °C for 4 days before microscopically determining virus-related cytopathic effects (CPE) such as cell rounding, detachment, degeneration, and syncytium formation. Wells with a clear cytopathic effect of more than 10% of that of the virus control well (cells + virus) were determined as positive. Wells with no CPE were classified negative.

#### 2.4.3. Pseudovirus Assay to Determine SARS-CoV-2 Neutralizing Activity (PVN)

Lentivirus-based pseudovirus expressing the SARS-CoV-2 Wu01 spike protein (EPI_ISL_40671) was produced in 293-T cells FuGENE-6 transfection reagent (Promega) and supernatants were harvested and stored at −80 °C. For testing SARS-CoV-2 neutralizing activity, serial dilutions of serum (heat inactivated at 56 °C for 45 min) were co-incubated with pseudovirus supernatants for 1 h at 37 °C and thereafter, 293T cells engineered to express ACE2 were added [25]. After 48 h of incubation at 37 °C and 5% CO_2_, luciferase activity was determined after addition of luciferin/lysis buffer (10 mM MgCl_2_, 0.3 mM ATP), 0.5 mM Coenzyme A, 17 mM IGEPAL (all Sigma-Aldrich, St. Louis, MO, USA), and 1 mM D-Luciferin (GoldBio, St. Louis, MO, USA) in Tris-HCL) using a microplate reader (Berthold). After subtracting background relative luminescence units (RLUs) of un-infected cells, the 50% Inhibitory dose (ID50) was determined as the serum dilution with 50% RLU reduction compared to untreated virus control wells. Every serum sample was measured on different days in two independent experiments, and the mean ID50 values are presented.

### 2.5. Statistical Analysis

Continuous variables were expressed as median (interquartile range, IQR) or mean ± standard deviation (SD) and compared using the Wilcoxon rank sum test or the unpaired Student’s *t*-test. Categorical variables were compared using either the χ^2^ test or the Fisher exact test, as appropriate. The Spearman rank correlation coefficient ρ was calculated as a measure of strength of the relationship between serological assay outcomes and the pseudovirus neutralization assay. The correlation between the 3-level ordinal live virus neutralization assay outcome and categorized binary outcomes of serologic assays was evaluated by calculating the Kendall’s coefficient of rank correlation τ. Additionally, two neutralizing cutoff titers were assessed for their concordance with the binary outcomes of the commercial serological assays using the Cohen’s κ. Two-sided *p*-values were presented, and an α of 0.05 was determined as the cutoff for significance. All statistical analyses were performed using R (version 3.6.3, R Foundation for Statistical Computing, Vienna, Austria).

## 3. Results

### 3.1. Sensitivity of Immundiagnostik IDK^®^ Anti-SARS-CoV-2 S1 IgG Assay in Comparison with Four Commercially Available Serological Tests and Two Virus-Neutralization Immunoassays in a Cohort with Previous SARS-CoV-2 Infection (Group A)

**Figure 2:**
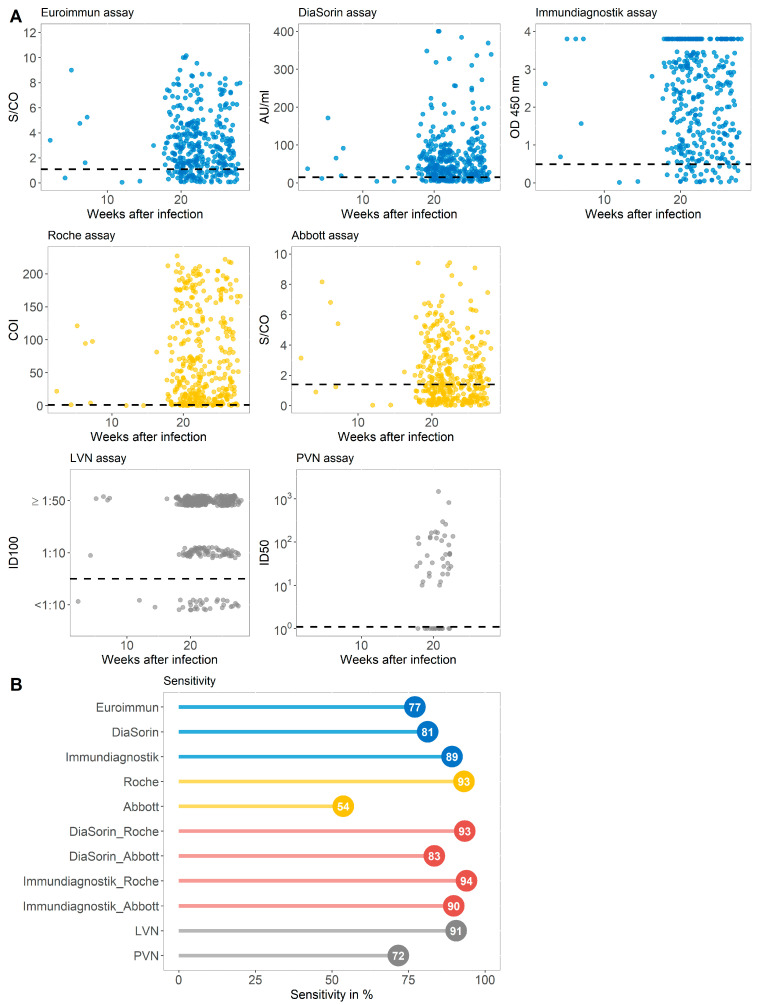
(**A**) Antibody values of five commercially available immunoassays targeting the S (spike) (blue) or the NC (nucleocapsid) protein (yellow) and virus neutralizing assays (grey) displayed against the weeks after infection for each individual from group A. Dashed horizontal lines display cutoff values of individual serological assays. Results of the virus neutralizing immunoassay are categorized into <1:10, 1:10, and ≥1:50. (**B**) The sensitivity in % achieved by assays against the S-protein (blue), the NC antigen (yellow), the combination of assays targeting the different proteins (red), and the virus neutralizing (VN) test (grey) for group A. LVN, live virus neutralization assay; PVN, pseudovirus neutralization assay; ID100, 100% inhibitory dilution; ID50, 50% inhibitory dose.

To evaluate the general sensitivity of serological tests with different antigen targets, we compared three commercially available immunoassays targeting the S-protein, two assays detecting the NC protein, a combination of assays targeting different antigens, and two VN assays (Supplementary Table S2). Serum samples of 363 convalescent patients with prior SARS-CoV-2 infection collected between August and September 2020 (group A) were used for this evaluation (Figure 2A). In this group, 5.9% of the individuals declared themselves as asymptomatic, 89.8% participants had a mild course of disease, while 4.33% of the subjects were hospitalized because of COVID-19 [16]. The median time between infection and antibody determination was 154 days (Table 2). As indicated in Figure 2B, the sensitivities achieved by immunoassays targeting the S-protein ranged between 77.1% and 89.2%. The assay by Immundiagnostik achieved a sensitivity of 89.2%. The serological tests detecting the NC antigen differed more strongly. While the test by Roche achieved the overall highest sensitivity (93.1%) of all assays, the Abbott immunoassay reached 53.7% sensitivity in this general cohort of recovered individuals. A combination of the best performing immunoassays (Immundiagnostik and Roche) with different protein targets resulted in a sensitivity of 93.9%. Of note, the sensitivity of 91% achieved by the LVN assay was below the sensitivity of the commercially available Roche test but higher than all the S-protein-targeting tests. A total of 342 (94.2%) participants with past SARS-CoV-2 infection in group A were tested antibody positive in at least one of the immunoassays evaluated.

**Figure 3:**
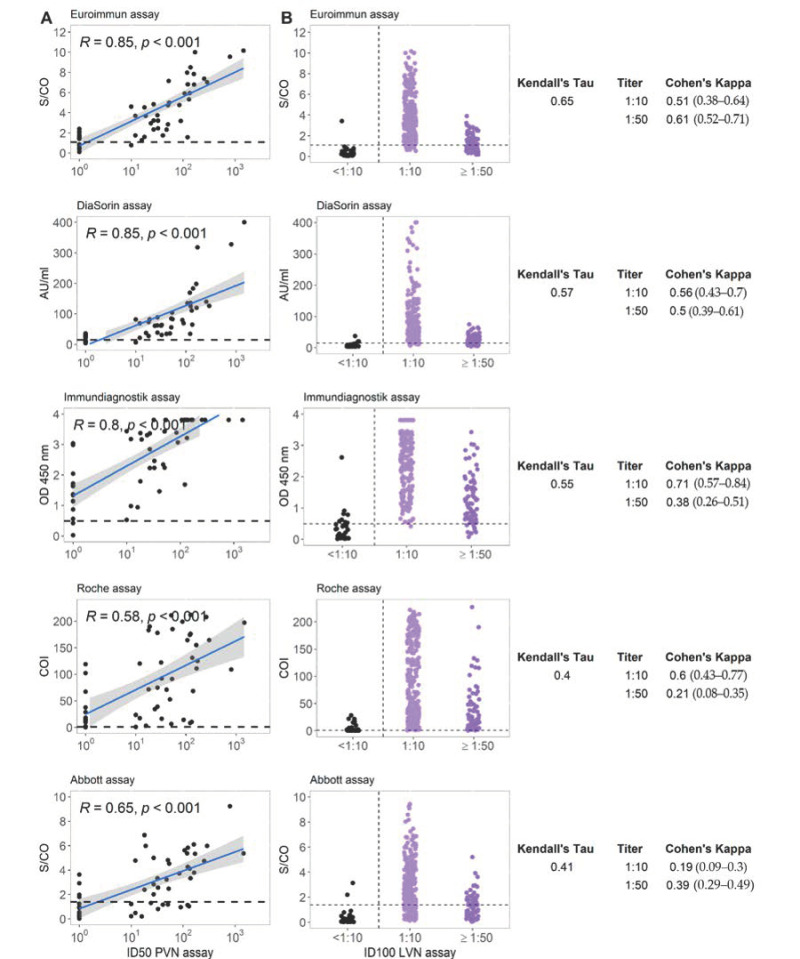
(**A**) Correlation between five commercial anti-SARS-CoV-2 serological assays and a pseudovirus neutralizing antibody titer for group A. R represents the Spearman rank correlation coefficient ρ. (**B**) Correlation between five commercial anti-SARS-CoV-2 serological assays and live virus neutralizing antibody titer for group A. Horizontal lines represent cutoff values for individual commercial tests. Kendall’s τ and Cohen’s κ are displayed for each test combination. LVN, live virus neutralization assay; PVN, pseudovirus neutralization assay; ID100, 100% inhibitory dilution; ID50, 50% inhibitory dose.

Figure 3A displays the correlation between the different commercial immunoassays and pseudovirus neutralization assay (PVN). The results of immunoassays targeting the S protein overall strongly correlated with the results of PVN (Spearman rank correlation coefficient ρ ranging between 0.80 and 0.85). In contrast to this, the correlation in case of assays targeting the NC protein was less strong with rho = 0.58–0.65. The relation between live virus neutralization assay (LVN) results and those from the different commercial immunoassays are shown in Figure 3B. The Kendall’s τ between LVN and the serological assay ranges between 0.40 and 0.65 with higher values for the assays targeting S-protein antigens. Cohen’s κ as a measure of concordance between neutralizing titer cutoffs and binary results of serological assays ranged widely. The Immundiagnostik assay reached a value of 0.71 at a LVN-cutoff titer of 1:10.

### 3.2. Sensitivity of Immundiagnostik IDK^®^ Anti-SARS-CoV-2 S1 IgG Assay and Three Commercially Available Serological Tests in a Cohort of COVID-19 Convalescent Subjects That Were Primarily Tested S1 IgG Non-Reactive (Group B)

Between April and July 2020, 169 individuals with confirmed prior SARS-CoV-2 infection attending our clinic were classified as non-reactive in the routine IgG screening using the Euroimmun S1 IgG ELISA (group B). In this group 16.0% of the subjects were asymptomatic, 80.0% of participants had a mild course of the disease, and 4.0% of the subjects were hospitalized because of COVID-19 [16]. The median time interval between infection and antibody determination was 47 days (Table 1).

**Figure 4:**
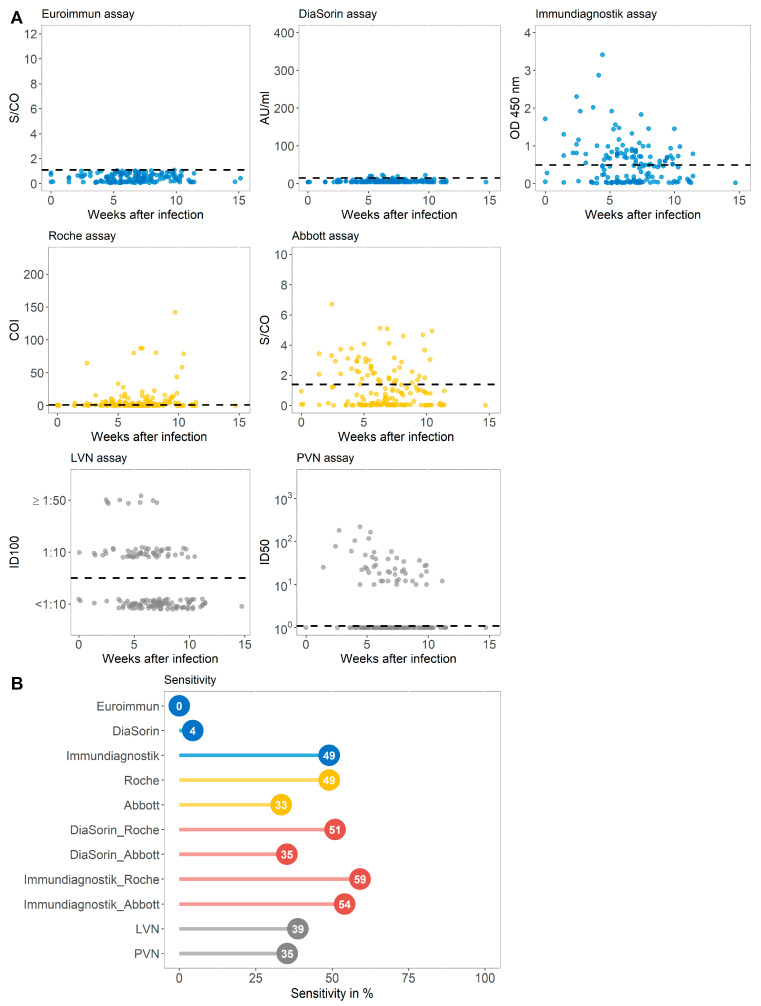
(**A**) Antibody values of five commercially available immunoassays targeting the S (spike) (blue) or the NC (nucleocapsid) protein (yellow) and virus neutralizing assays (grey) displayed against the weeks after infection for each individual from group B. Dashed horizontal lines display cutoff values of individual serological assays. Results of the virus neutralizing immunoassay are categorized into <1:10, 1:10, and ≥1:50. (**B**) The sensitivity in % achieved by assays against the S-protein (blue), the NC antigen (yellow), the combination of assays targeting the different proteins (red), and the VN test (grey) for group B. LVN, live virus neutralization assay; PVN, pseudovirus neutralization assay; ID100, 100% inhibitory dilution; ID50, 50% inhibitory dose.

By retesting the samples with two other assays against the S-protein, two commercially available immunoassays targeting the NC protein, different test combinations, and two VN assays, we investigated which serologic test would perform best in detecting IgG seropositive individuals in this population of individuals classified as S1 IgG non-reactive (Figure 4A and Supplementary Table S3).

While the DiaSorin immunoassay detected 7 patients as IgG positive (4.4%), the test by Immundiagnostik found 49.06% of this cohort to be IgG reactive. The same number was found to be antibody positive using the Roche test. Notably, when combining the Immundiagnostik and Roche tests, 94 out of 159 patients (59.1%) were detected as seroconverted (Figure 4B). Using two different virus neutralizing immunoassays, 38.8% and 35.3% of the initially IgG-negative participants were tested positive for the presence of neutralizing antibodies. A total of 97 (61%) individuals with prior SARS-CoV-2 infection but undetectable IgG antibodies (group B) were classified as seropositive in at least one of the other assays evaluated.

**Figure 5:**
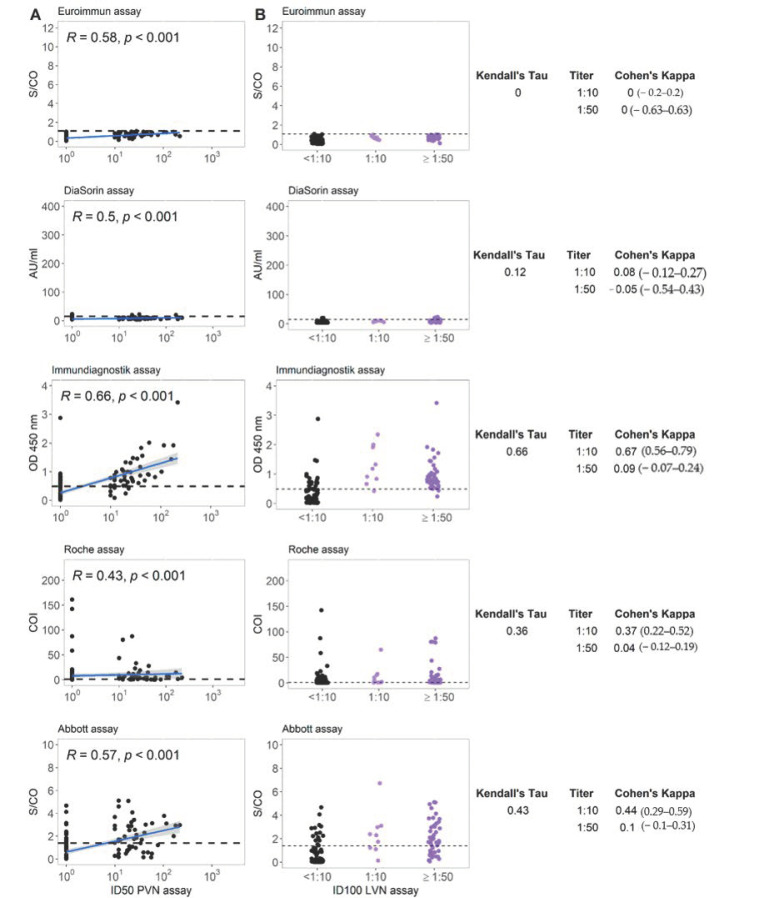
(**A**) Correlation between five commercial anti-SARS-CoV-2 serological assays and the pseudovirus neutralizing antibody titer for group B. R represents the Spearman rank correlation coefficient ρ. (**B**) Correlation between five commercial anti-SARS-CoV-2 serological assays and live virus neutralizing antibody titer for group B. Horizontal lines represent cutoff values for individual commercial tests. Kendall’s τ and Cohen’s κ are displayed for each test combination. LVN, live virus neutralization assay; PVN, pseudovirus neutralization assay; ID100, 100% inhibitory dilution; ID50, 50% inhibitory dose.

The correlation between the different commercial immunoassays and the pseudovirus neutralization assay (PVN) for the special sub-cohort of apparently non-responders is displayed in Figure 5A. The correlation between PVN and all evaluated commercial assays is moderate with a Spearman rank correlation coefficient ρ ranging between 0.43 in case of the Roche assay and 0.66 for the assay by Immundiagnostik. The relation between the neutralization assay using live virus results and those from the different commercial immunoassays in case of group B is shown in Figure 5B. The Kendall’s τ between the ordinal VN assay and the categorized serological tests ranges largely between 0.12 and 0.66 with the highest correlation observed with the Immundiagnostik assay. The correlation coefficients between the LVN assay and the serological assays targeting the NC protein do not differ profoundly from those measured in the unbiased convalescent cohort. The highest concordance as determined by the Cohen’s κ was observed at an LVN-titer of 1:10 with the serologic assay of Immundiagnostik (κ = 0.67) (Figure 5B).

### 3.3. Specificity of Immundiagnostik IDK^®^ Anti-SARS-CoV-2 S1 IgG Assay in Comparison with Four Commercially Available Serological Tests in a SARS-CoV-2 Negative Control Group (Group C)

**Figure 6:**
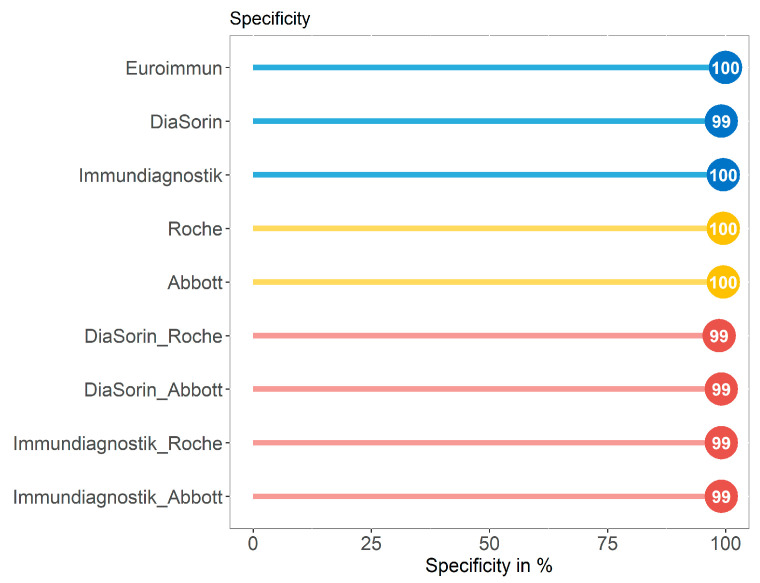
The specificity achieved by assays against the S (spike) protein (blue), the NC (nucleocapsid) antigen (yellow), and the combination of assays targeting the different proteins (red) for group C.

We compared the performance in terms of specificity of the Immundiagnostik assay in comparison with four serological tests with different antigen targets using a control group (group C, *n* = 227) with 177 serum samples of individuals without suspected SARS-CoV-2 infection collected in early 2020 and 50 serum samples that were collected in 2019 in our clinic. All assays or a combination of those achieved a specificity of 99–100% (Figure 6 and Supplementary Table S4).

## 4. Discussion

The detection of specific antibodies against defined infectious pathogens is commonly used as a marker of infection or immunity. The determination of the serological immune status against hepatitis B virus or measles are widely known examples of tests to evaluate individual protection against these agents [26]. However, in the case of SARS-CoV-2, it was observed that high proportions of patients can remain seronegative even months after infection or that antibody levels wane over time, particularly in patients with asymptomatic or mild courses of disease [27,28,29,30,31]. Considering that the majority of COVID-19 cases have a course of the disease with only mild symptoms, it is of outstanding importance to well-characterize individuals with low or undetectable serum IgG response and investigate if non-detection is equal to absence or just a result of less sensitive laboratory assessment methods [12].

In the present study, we evaluated the performance of a novel ELISA commercial assay approved for the detection of anti-SARS-CoV-2 IgG, targeting epitopes within the S1 region in patients recovered from mostly mild COVID-19. In particular, we studied two different sub-cohorts of convalescent individuals, one unbiased group of recovered patients and one group of individuals that were primarily tested S1 IgG non-reactive.

The evaluation of serologic immunoassays in the general cohort of individuals with previous SARS-CoV-2 infection unraveled varying test performances. In line with other works, tests targeting the S-protein ranged between a sensitivity of 77.1% and 89.2% [32]. Differently from previous observations, we detected higher differences for assays targeting NC with a sensitivities ranging between 53.7% and 93.1% [33].

In group B, defined as individuals with undetectable IgG antibodies using a commercial S1 IgG ELISA despite prior SARS-CoV-2 infection confirmed by RT-PCR, we detected a high proportion of patients that produced specific anti-SARS-CoV-2 IgG antibodies. Whereas the assay by DiaSorin detected IgG in 4% of these patients, the Abbott assay was able to detect antibodies in 33%. The tests by Immundiagnostik and Roche both found 49.1%. When combining these two immunoassays with the highest detection rate, this proportion reached 59.1%. 

The moderate performance of the Alinity Abbott assay in our study is in line with the results by Muecksch et al. [10]. The authors reported a decay of the sensitivity from >90% within the first 40 days post-infection to 71% in samples tested more than 80 days after diagnosis, whereas Roche Elecsys^®^ titers stayed more stable overtime. Similarly, in the present study, the Abbott assay was observed less sensitive in comparison to the other commercial assays when used investigating individuals at a median time of 154 days post-infection (group A). In contrast to this, the difference between the Abbott and the Roche assays was less prominent in case of group B. This group was tested for the presence of antibodies after a median time of 47 days after SARS-CoV-2 infection. Muecksch et al. suggested that the different performance of the two NC assays may be attributable to the antigen bridging approach characterizing the Roche assay.

Overall, these data confirmed previous conclusions that disease severity and time since infection can critically impact on the declared sensitivity of commercial SARS-CoV-2 serological assays [20]. Furthermore, the S-protein appears to be a more reliable target than the NC region [34].

Our data suggest that the performance of the Immundiagnostik assay in terms of sensitivity is similarly high compared to the assay by Roche in both subgroups. 

Among the S-protein based assays, Immundiagnostik achieved the highest sensitivity. A potential explanation could be that different parts of the S-protein from SARS CoV-2 were used. For the development of the Immundiagnostik ELISA kit, three different regions of the S-protein were tested during test development. The most robust signal with low background noise and high reproducibility was obtained with the N-terminal part of the S1-protein. For the set-up, over 762 plasma samples, collected between 2017 and 2018, were tested to define a tight cut off (Extended Material and Methods, Supplementary Materials Figure S1). In comparison to the Euroimmun S1 protein, the Immundiagnostik protein is 156 amino acid shorter and the common region differs by four amino acids.

Overall, neutralizing antibodies are considered one of the main parameters to measure protective immunity against SARS-CoV-2, although their role in the case of acute disease is controversial [35,36,37,38]. Thus, the detection of neutralizing antibodies could play a key role in monitoring vaccine efficacy, but since biosafety requirements are high, it is of critical interest that new serologic immunoassays are not only more sensitive, but also correlate well with neutralization assays [39,40,41]. Recent studies indicated that commercial assays targeting the S-protein often correlate better with neutralizing antibody titers than those targeting the NC protein [42,43]. Likewise, our presented data suggest that immunoassays targeting the S antigen correlated to a higher degree with results from the virus neutralizing assay than those targeting the NC protein. In group B, the serologic test by Immundiagnostik achieved high correlation with results from the virus neutralizing assay, and the concordance was highest if low neutralizing titers of 1:10 were considered as cutoff values for positivity. 

SARS-CoV-2 specific immunological memory is not necessarily mirrored by antibody levels detected in serological assays. As recently shown, persistence and prolonged affinity of SARS-CoV-2 reactive B cells is observed in individuals after recovering from COVID-19 [44,45,46]. By detecting a high percentage of seropositive patients at the median time of 154 days post infection, our data support the findings of a more durable immune response upon SARS-CoV-2 infection. However, although the proportion of IgG seropositive individuals appears to be higher if tested with more sensitive immunoassays, the potential of these low antibodies to protect from COVID-19 remains to be further investigated. 

## 5. Conclusions

Our study has two main outcomes. Firstly, we found the serological assay by Immundiagnostik detecting SARS-CoV-2 S1-directed IgG antibodies to be of high sensitivity and specificity. Its combined use with the assay by Roche could be useful option in order to differentiate infected from vaccinated individuals. Secondly, compared to assays detecting anti-NC antibodies, assays targeting the SARS-CoV-2 S protein, including the test by Immundiagnostik, correlated more strongly with serum neutralizing activity. 

## Figures and Tables

**Table 1:** Characteristics of evaluated commercial serological assays for detection of anti-SARS-CoV-2 antibodies.

Company	Assay	Method	Platform	Target
Abbott	SARS-CoV-2 IgG	CMIA	Alinity I (Abbott)	NC
DiaSorin	LIAISON^®^ SARS-CoV-2 S1/S2 IgG	CLIA	Liaison XL (DiaSorin)	S1/S2
Euroimmun	Anti-SARS-CoV-2 IgG	ELISA	Euroimmun Analyzer I (Euroimmun)	S1
Immundiagnostik	IDK^®^ anti-SARS-CoV-2 IgG	ELISA	DYNEX DSX (Dynex Technologies)	S1
Roche	Elecsys^®^ anti-SARS-CoV-2 pan-Ig	ECLIA	Cobas 8000 (Roche)	NC

NC, nucleocapsid; S1, spike protein subunit 1; S2, spike protein subunit 2; CMIA, chemiluminescent microparticle immunoassay; CLIA, chemiluminescent immunoassay; ECLIA, electrochemiluminescent immunoassay; ELISA, enzyme-linked immunosorbent assay.

**Table 2:** Demographical and clinical characteristics of participants.

Parameters	Group A, *n* = 363	Group B, *n* = 169
Female, *n* (%)	200 (55.24)	103 (60.95)
Male, *n* (%) *	162 (44.75)	66 (39.05)
Age in years, mean ± SD	44.09 ± 12.86	42.69 ± 12.86
Days after disease onset, median (IQR)	154 (141–176)	47 (35–56)
Asymptomatic, *n* (%)	15 (5.90)	20 (16.00)
Mild, *n* (%)	228 (89.77)	100 (80.00)
Severe, *n* (%) **	11 (4.33)	5 (4.00)

SD, standard deviation; IQR, interquartile range; * no data available for 1 subject in group A; ** no data available for 109 subjects in group A and 44 subjects in group B.

## Data Availability

All observation data are accessible via the Supplementary Materials.

## Supplementary Materials

The following are available online at https://www.mdpi.com/article/10.3390/microorganisms9040733/s1, Extended Materials and Methods: Supplementary information on the establishment of a new SARS-CoV-2 serological test; Figure S1: Discrimination between anti-SARS-CoV-2 IgG negative and positive samples with two different constructs of the S1 protein used as an antigen; Table S1: Dataset; Table S2: Sensitivity of five commercially available serological tests, combination of tests, and two virus neutralizing immunoassays in a cohort with previous infection with SARS-CoV-2.; Table S3: Sensitivity of four commercially available serological tests, combination of tests, and two virus neutralizing immunoassays in a cohort with previous infection with SARS-CoV-2 but undetectable IgG antibodies by the Euroimmun assay; Table S4: Specificity of five commercially available serological tests and combination of tests in a SARS-CoV-2 negative control group.
