# Supplementary material for: Evaluation of a New Spike (S)-Protein-Based Commercial Immunoassay for the Detection of Anti-SARS-CoV-2 IgG
Source: Microorganisms. 2021 Mar 31;9(4):733. doi: 10.3390/microorganisms9040733 (PMC8067155; doi:10.3390/microorganisms9040733)
Supplement: Supplementary file 1 [file microorganisms-09-00733-s001.zip › Supplementary Table S2-4, Figure S1.pdf]

Supplementary Materials

Extended Materials and Methods –

Supplementary information on the establishment of a new SARS-CoV-2 serological test:

The OD of the cut-off control was set based on a set of predefined and well characterized samples. First, the upper limit of the value was set using serum samples from patients with a previous SARS-CoV-2 infection confirmed by PCR. Second, the lower limit was defined by using serum samples obtained between 2017 and 2019 as well as serum samples from patients with negative SARS-CoV-2 PCR results. These samples were used to identify unspecific signals. Next, the cut-off control was optimized and adjusted with two small samples sets: false positive samples identified with competitors' assays and samples positive for other coronaviruses, e.g. HKU-1. Lastly, the OD value of the cut-off control was confirmed using a large set of serum samples and data, as published in the IFU, for sensitivity and specificity were obtained.

The three different S-protein constructs were designed and produced at the University of Cologne (in laboratory of Manuel Koch). The development of the ELISA Kit was then entirely performed by Immundiagnostik. To choose the right antigen as base for the assay three different constructs of the S1 protein were tested with the same subsets of serum samples. Serum samples were obtained from patients who underwent a SARS-CoV-2 infection, which was confirmed by RT-PCR or collected before 2020. While further testing with one of the three constructs was early stopped after false negative results were obtained (serum samples from patients with positive PCR results), two constructs (S1 truncated and S1 full length) showed similar results in preliminary testing. Therefore, we continued with these two candidates. As the aim was to develop a test, which is able to clearly differentiate between anti-SARS-CoV-2 IgG negative and anti-SARS-CoV-2 IgG positive samples, we tested the S1 full length and S1 truncated under different conditions. Finally, the best differentiation between positive and negative samples was achieved using the truncated version. A representative result of these tests is shown in the Supplementary Figure 1.

Supplementary Figure S1: Discrimination between anti-SARS-CoV-2 IgG negative and positive samples with two different constructs of S1 protein used as antigen

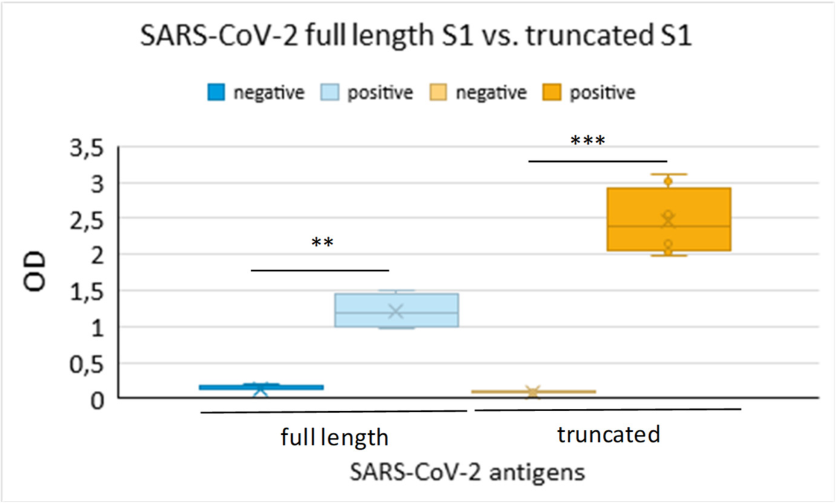

*Plates were coated either with S1 full length protein or with S1 truncated protein. Serum samples collected before 2020 were assumed as anti-SARS-CoV-2 negative (n=5) and serum samples from patients with RT-PCR confirmed SARS-CoV-2 infection were assumed to be positive for anti-SARS-CoV-2 IgG (n=11). (**p<0,01, ***p<0,001, Student's T-Test).*

Supplementary Table S2. Sensitivity of five commercially available serological tests, combination of tests, and two virus-neutralizing immunoassays in a cohort with previous infection with SARS-CoV-2.

	n	Pos/neg	Sensitivity in %
Euroimmun	363	280/83	77.13
DiaSorin	363	295/68	81.27
Immundiagnostik	362	323/39	89.23
Roche	363	338/25	93.11
Abbott	363	195/168	53.71
Euroimmun/Roche	363	338/25	93.11
Euroimmun/ Abbott	363	292/71	80.44
DiaSorin /Roche	363	339/24	93.39
DiaSorin / Abbott	363	303/60	83.47
Immundiagnostik/Roche	363	341/22	93.94
Immundiagnostik/ Abbott	363	326/37	89.81
Live virus neutralization test	351	318/33	90.60
Pseudovirus neutralization test	53	38/15	71.70

Supplementary Table S3. Sensitivity of four commercially available serological tests, combination of tests, and two virus-neutralizing immunoassays in a cohort with previous infection with SARS-CoV-2 but undetectable IgG antibodies by the Euroimmun assay.

	n	Pos/neg	Sensitivity in %
Euroimmun	159	0/159	0
DiaSorin	159	7/152	4.40
Immundiagnostik	159	78/81	49.06
Roche	159	78/81	49.06
Abbott	159	53/106	33.33
DiaSorin/Roche	159	81/78	50.94
DiaSorin/Abbott	159	56/103	35.22
Immundiagnostik/Roche	159	94/65	59.12
Immundiagnostik/Abbott	159	86/73	54.09
Live virus neutralization test	152	60/93	38.82
Pseudovirus neutralization test	136	88/48	35.29

Supplementary Table S4. Specificity of five commercially available serological tests and combination of tests in a SARS-CoV-2 negative control group.

	n	Pos/neg	Specificity in %
Euroimmun	227	0/227	100.0
DiaSorin	227	2/225	99.1
Immundiagnostik	226	1/225	99.6
Roche	227	1/226	99.6
Abbott	227	1/226	99.6
Euroimmun/Roche	227	1/226	99.6
Euroimmun/Abbott	227	1/226	99.6
DiaSorin /Roche	227	3/224	98.7
DiaSorin/Abbott	227	2/225	99.1
Immundiagnostik/Roche	226	2/224	99.1
Immundiagnostik/Abbott	226	2/224	99.1
